# A Bayesian hierarchical model with spatial variable selection: the effect of weather on insurance claims

**DOI:** 10.1111/j.1467-9876.2012.01039.x

**Published:** 2013-01

**Authors:** Ida Scheel, Egil Ferkingstad, Arnoldo Frigessi, Ola Haug, Mikkel Hinnerichsen, Elisabeth Meze-Hausken

**Affiliations:** 1 University of Oslo and Norwegian Computing CenterOslo, Norway; 2 Norwegian Computing CenterOslo, Norway; 3 University of Oslo and Norwegian Computing CenterOslo, Norway; 4 GjensidigeOslo, Norway

**Keywords:** Bayesian Poisson hurdle, Climate change, Generalized linear models, Hierarchical models, Spatial variable selection, Zero-altered Poisson model

## Abstract

Climate change will affect the insurance industry. We develop a Bayesian hierarchical statistical approach to explain and predict insurance losses due to weather events at a local geographic scale. The number of weather-related insurance claims is modelled by combining generalized linear models with spatially smoothed variable selection. Using Gibbs sampling and reversible jump Markov chain Monte Carlo methods, this model is fitted on daily weather and insurance data from each of the 319 municipalities which constitute southern and central Norway for the period 1997–2006. Precise out-of-sample predictions validate the model. Our results show interesting regional patterns in the effect of different weather covariates. In addition to being useful for insurance pricing, our model can be used for short-term predictions based on weather forecasts and for long-term predictions based on downscaled climate models.

## 1. Introduction

The global insurance industry is highly exposed to risks caused by weather-related events. There are clear signs of a significant increase in the number of claims in the past two decades, possibly due to changes in the spatial distribution, frequency and intensity of both ordinary and catastrophic weather events. Simultaneously, demographic and socio-economic trends are increasing society's exposure to weather-related losses. An analysis of insurance industry data shows that losses from catastrophic weather-related events (e.g. hurricanes, storms, floods and extreme droughts) have increased by 2% each year since the 1970s, adjusting for changes in wealth, inflation and population growth (Muir-Wood *et al.*, 2006). Whereas extreme and large-scale catastrophic events represent roughly 40% of insured weather-related losses globally, small-scale weather-related events (such as rain, hailstorms, heavy wind and frost) account for most incurred losses (Mills *et al.*, 2005; Botzen and van den Bergh, 2008). Losses due to non-catastrophic weather patterns are expected to increase non-linearly with intensity of precipitation. Neglecting this trend could lead to underestimating losses. Damage to buildings caused by precipitation has been investigated previously (Blong, 2004; Cornick and Dalgliesh, 2003; Schuster *et al.*, 2006; Sanders and Phillipson, 2003).

To understand patterns of risk over time and geography, we explore the relationship between weather events and incurred losses by analysing historical data. We analyse the relationship between the number of claims and weather data, using 10 years of daily insurance and meteorological data in Norway. To investigate associations between claims and weather, it is essential that the data show a fine spatial and temporal resolution. We focus on damage caused to privately owned buildings and exclude the small number of catastrophic weather-related events which, in Norway, are covered by a separate national fund.

In this paper we model only the number of claims, not their size. It is customary in the actuarial literature to condition on the frequency component when analysing joint frequency and size distributions (see for example Klugman *et al.* (2008)). As discussed in Frees and Valdez (2008), gamma models are appropriate for claim size (Haug *et al.*, 2011). It is also possible to develop a spatial model for claim size, but weather conditions produce a stronger spatial effect on claim numbers than on claim size. The severity of insurance claims is related to factors such as the wealth of the population, type of construction, age of structures and general economic factors (Department for Environment, Food and Rural Affairs, 2004).

This study aims

- to understand which weather patterns are responsible for claims and
- to predict the number of losses given a particular weather pattern.

In both cases we work at a local scale. Our results can be used to develop strategies to limit the effects of weather events, through preventive actions, in collaboration with insured customers and local authorities. Further, the results help to update risk estimation and premium calculations. Our model can be used to predict the number of claims at a regional scale, while it rains, by using realtime meteorological data. This is useful particularly when actual reporting is delayed. In addition, we provide short-term predictions of insurance losses based on weather forecasts 1 or 2 weeks in advance. Insurance companies can use these predictions to organize inspections and support in the right place immediately for clients whose property is damaged.

We can also use the model to investigate the effect of hypothetical weather scenarios, constructed, for example, by resampling historical events and placing them at different geographical locations. More interestingly, one can use downscaled climate models to understand the potential exposure of an insured portfolio under future climate conditions.

We cast the problem in a Bayesian space–time setting, where appropriate regressions are performed in each municipality, with weather variables as covariates. We wish to select the relevant covariates locally but assume that the local model varies smoothly over the geography of Norway, as we do not expect abrupt changes in weather-related claims in areas with comparable geographical conditions. This leads to the formulation of a hierarchical Bayesian spatial variable selection process. Inference can be conveniently split into two separate tasks: one for the Bernoulli probability of a claim; the other for the intensity of the positive Poisson count. Our results show interesting regional patterns in weather covariates contributing to the presence or absence of claims and to the number of claims, and out-of-sample predictions are sufficiently precise.

There has been some research on the relationship between weather and the insurance industry (Vellinga *et al.*, 2001; Nordhaus, 2008; Association of British Insurers, 2005), but this area lacks public data owing to their presumed competitive value; as a result, studies are scarce. Some data aggregated in space and time are available, and Mills (2005) identified financial services and asset management companies as vulnerable to climate change. Botzen and van den Bergh (2008) discussed various types of risk exposure to weather-related events in the insurance industry, concentrating on the case of the Netherlands. We support Botzen and van den Bergh's (2008) call for the industry to share their data. The study that is presented in this paper shows that insight can be gained via thorough statistical analysis. Adaptation to climate change for the insurance industry was discussed in Warner *et al.* (2009) and Kleindorfer (2010). Actuarial applications of hierarchical insurance claim models, including several zero-inflated stochastic models, were presented in the fundamental papers Frees and Valdez (2008), Frees *et al.* (2009), Boucher and Denuit (2006) and Boucher and Guillen (2009). A few references explore spatial models for insurance data (Taylor, 1989; Boskov and Verrall, 1994; Dimakos and Frigessi, 2002).

This paper proceeds as follows. In Section 2, we describe our data set. Section 3 sets forth the hierarchical model. We present results in Section 4 and conclude with a brief discussion in Section 7.

## 2. Insurance and weather data

Insurance data are from Gjensidige (http://www.gjensidige.no), the largest non-life insurance company in Norway, and include all insured private buildings in the period 1997–2006. For a more complete description of the data, see Haug *et al.* (2008, 2011). We focus on the municipalities of central and southern Norway, which generate the majority of claims. For each of the *K*=319 municipalities, we obtain the daily number of claims due to damage caused by either precipitation, surface water, snow melt, undermined drainage, sewage back-flow or blocked pipes. We also have the monthly number of insured buildings for each municipality, representing exposure. For municipality *k*,*k*=1,2,…,*K*, *N*_*kt*_ is the observed number of claims on day *t*, *T*_*k*_ is the set of days for which we observe *N*_*kt*_ (as there are a few missing values in the data) and *t*_*k*_ is the number of days in *T*_*k*_. Let 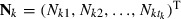 be the vector of claims and 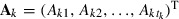 be the vector of insured units in municipality *k* for each time point.

**Fig. 1:**
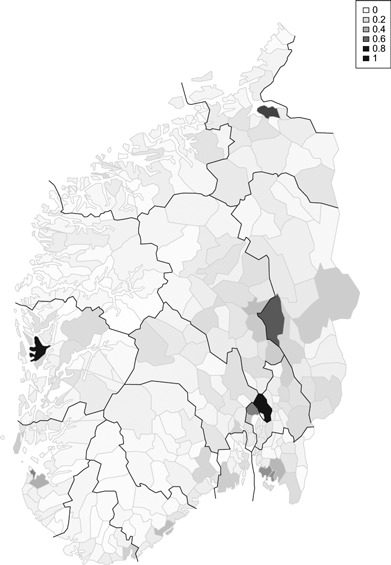
Exposure for each municipality in central and southern Norway described by the average daily number of insurance policies as a percentage with respect to the maximum (Bergen)

Fig. 1 describes the spatial variability of the exposure: for each municipality, we compute the average daily number of policies and plot the percentage with respect to the maximum exposure (which is in Bergen). Most claims are concentrated in the main cities. The mean claim size in the various municipalities ranged from 20000 NOK to 65000 NOK (price index adjusted; the data are not shown).

The Norwegian Meteorological Institute (http://www.met.no), together with the Norwegian Water Resources and Energy Directorate (http://www.nve.no), produce the meteorological and hydrological data: daily mean precipitation, mean temperature, drainage run-off and snow water equivalent for each municipality, on each day, for the period 1997–2006. Data are interpolated from a grid of monitoring stations, weighting areas within each municipality proportionally to population density. In this way, the meteorological covariates describe more accurately the weather events at locations of insured buildings for larger municipalities.

Table 1 shows the *q*=7 covariates that we use in our models; four are basic and three derived. The measurement period for precipitation is delayed by 6 h compared with calendar time, i.e. the total precipitation registered in day *t*+1 is the amount collected from 6 a.m. of day *t* to 6 a.m. of day *t*+1. *R*_3*t*_ is the accumulated rain over the preceding 3 days. The difference in snow water equivalent for successive days, *S*_Δ_, can also be relevant. We define **X**_*k*_ as the *t*_*k*_×*q* weather covariate matrix for municipality *k*. We use the full covariate matrix as well as a reduced version corresponding to days with positive numbers of claims, both of which are appropriately centred and scaled.

**Table 1:** Weather variables directly observed and derived

*Variable*	*Description*	*Unit*
*Observed*		
*R*_*t*_	Precipitation registered day *t* (mainly collected during day *t*−1)	mm
*C*_*t*_	Mean temperature in day *t*	^∘^C
*D*_*t*_	Total drainage run-off in day *t*	mm
*S*_*t*_	Total snow–water equivalent in day *t*	mm
*Derived*		
*R*_*t*+1_	Precipitation registered day *t*+1 (mainly collected during day *t*)	mm
*R*_3*t*_	Sum of precipitation in last 3 days	mm
	( 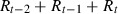 )	
*S*_Δ_	Change in snow–water equivalent ( 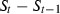 )	mm

The choice of covariates is based on insurance company experience (e.g. 3 days’ accumulated water and change in snow–water difference, as a potential risk indicator for water seeping from the ground into the basement, damaging foundations and interior), evidence from other studies (e.g. Blong (2004)) and availability of the same covariates as outputs of downscaled climate models, for future scenario simulation.

## 3. Bayesian Poisson hurdle model with Ising smoothed variable selection

The daily number of claims *N*_*kt*_ in a municipality *k* is 0 more often than would be modelled with a Poisson distribution, which is often the case for insurance count data. Further, we expect a threshold effect of the weather covariates on claims, as no damage is caused by normal weather states. Different weather patterns are thought to be responsible for having a day with no claims as opposed to one or more claims, and for the actual count on days with claims. This leads to the hurdle model, as in Mullahy (1986) and Heilbron (1994), rather than, for example, a zero-inflated Poisson model (Lambert, 1992). This model consists of two parts: one part is a Bernoulli distribution modelling whether the count is 0 or positive; the second models strictly positive counts by a count distribution.

Let *α*_*kt*_ be the probability of a 0 count. The latent binary variable *ζ*_*kt*_ indicates whether there is a 0 (*ζ*_*kt*_=0) or positive (*ζ*_*kt*_=1) count, with an *a priori* Bernoulli(1−*α*_*kt*_) probability. The second component of the hurdle model, modelling positive counts, is assumed to be positive Poisson with parameter *λ*_*kt*_. We call the whole model the Bayesian Poisson hurdle (BPH) model. The BPH model for the number of claims is hence


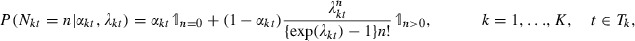
(1)

where 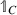 equals 1 when *C* is true and 0 otherwise, and we write 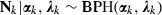, where ***α***_*k*_ is the vector of *α*_*kt*_ for all *t*, and ***λ***_*k*_ is the vector of *λ*_*kt*_ defined only on days with positive counts. We model *α*_*kt*_ and *λ*_*kt*_ by generalized linear models (GLMs), separately for each municipality, but with spatially smoothed variable selection. We use a logit link for *α*_*kt*_ in the Bernoulli distribution, and a log-linear model for *λ*_*kt*_ in the positive Poisson distribution, with Gaussian overdispersion. Each municipality has a pair of GLMs for *α*_*kt*_ and *λ*_*kt*_. A consequence of the hurdle model formulation is that *λ*_*kt*_ only matters for day *t* and municipality *k* if there is a positive count. Consequently *λ*_*kt*_ is dependent on *ζ*_*kt*_ and *α*_*kt*_. However, posterior inference for the model divides into two parts: the 0 count and the positive count. These can be run completely separately, as they are conditionally independent given the data. We first describe the GLM model for *λ*_*kt*_, and then the GLM model for *α*_*kt*_.

To model the selection of covariates appropriate for the GLM for ***λ***_*k*_ for municipality *k*, we introduce the vector of binary variables 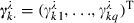. For municipality *k* and covariate *j*, 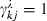 means that covariate *j* enters the model for ***λ***_*k*_, and 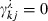 otherwise. Let 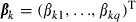 be the coefficients of the covariates for the GLM for *λ*_*kt*_, with *β*_*kj*_=0 if 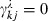. We define the reduced vector 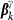 as the vector of *β*_*kj*_ for which 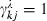. The intercept *β*_*k*0_ is always part of the model. We reduce **X**_*k*_ to include only the rows corresponding to the days with positive counts, and for convenience denote this, still, as **X**_*k*_, with a slight abuse of notation. Furthermore, we define 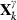 as the reduced covariate matrix consisting only of the columns *j* of **X**_*k*_ for which 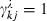. The GLM for ***λ***_*k*_ is given by


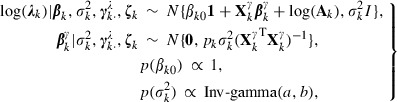
(2)

where *p*_*k*_ is the number of days with *N*_*kt*_≥1 and **A**_*k*_ is the vector of insured units in municipality *k* for days when claims occur. **A**_*k*_ enters the model as a constant term, which is equivalent to modelling the number of claims per insured unit.

The structure of the covariance of 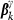 is in the form of a *g*-prior (Zellner, 1986) and has been considered for variable selection in Gaussian linear models (see for example Smith and Kohn (1996), George and McCulloch (1997) and Fernandez *et al.* (2001)). In such models, the *g*-prior covariance structure replicates the covariance structure of the likelihood. The Gaussian–inverse gamma conjugate prior structure is convenient, as it allows calculation of the marginal likelihood for the variable selection parameters, thus enabling sampling of the variable selection indicator variables directly. This avoids the difficulty of the variable dimension of the parameter space. In our case, the Gaussian linear regression is lifted one level up in the hierarchy to model log (*λ*_*kt*_), which itself is a parameter in the Poisson hurdle model. Thus, the *g*-prior does not mimic the covariance structure of the likelihood, but it has the same convenient properties. Our choice of *p*_*k*_ (the number of observations for municipality *k*) as the scale factor corresponds to unit prior information for 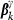 as in Kass and Wasserman (1995), Kohn *et al.* (2001) and Smith and Fahrmeir (2007).

We make the variable selection for covariate *j* smooth across Norway by assuming, *a priori*, a spatial model for ***γ***^*λ*^. In this way, we can borrow strength due to inhomogeneous exposures, which facilitates variable selection for areas with little exposure and data. We define the *K*×*q* matrix of binary indicator random variables:


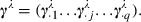


We assume a spatial model for each covariate *j* across Norway, by giving 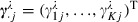 (the vector of indicators for covariate *j* over all municipalities *K*) an Ising prior distribution


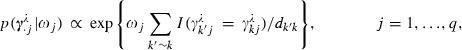


with *ω*_*j*_*a priori* uniformly distributed on (0,*ω*_max_) for some fixed value of *ω*_max_. Here, *k*^′^∼*k* indicates that the two municipalities *k* and *k*^′^ are neighbours, with a distance of 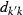. Various topologies are possible. Here, we simply assume that municipalities sharing a boundary are neighbours, and 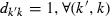. The parameter *ω*_*j*_ controls the level of smoothness of covariate *j*, but not the probability that the covariate enters the model in a location. The *q*-variable-selection indicator variable vectors 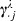, *j*=1,…,*q*, are assumed *a priori* to be independent. Smith and Fahrmeir (2007) used the Ising model to smooth spatially the variable selection process in linear regression models on a regular lattice. In their application, with many covariates, it was important to reduce *a priori* the chances for a covariate to be selected. This is achieved with an external field in the Ising model. In our case, this would then be


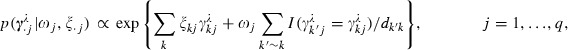


where the coefficients *ξ*_*kj*_>0 for some municipality *k* or in certain geographical areas would imply *a priori* that the probability that covariate *j* should be selected is larger than 0.5, which is in fact a specific function of *ξ*_*kj*_ which can be computed. In our application we have no reason to assume that a certain covariate should *a priori* be favoured to enter the model. All the seven covariates were chosen by experts, because it is *a priori* conceivable that they affect the number of claims. There is no further knowledge on specific regions where certain covariates should *a priori* enter the model. Therefore, we must assume a non-informative prior, where *a priori* the probability that each covariate enters the model in any location is 0.5. Our Ising model must have no external field *a priori*. In the posterior model, the likelihood will act as an external field, so that claim and covariate data will automatically act as an external field, tilting the model towards covariate selection. If we were to run our model with an external field in the Ising prior for a certain covariate, then the same covariate would simply enter the model *a posteriori* more often. We can imagine that in other situations, where *a priori* knowledge about the effect of specific weather conditions on an outcome is available, an *a priori* external field would be very useful.

We move now to the other component of the model, describing the presence or absence of claims. The variable selection in the GLM for *α*_*kt*_ is done in the same way as for ***λ***_*k*_, using the variable selection indicator matrix ***γ***^*α*^. For each municipality *k*, 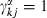 means that covariate *j* enters the model for *α*_*kt*_. We now let 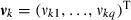 be the vector of regression coefficients for the GLM for *α*_*kt*_ and define 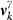 as the reduced vector of *ν*_*kj*_ for which 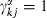. Here, **X**_*k*_ is again the full covariate matrix for all the days *t* ∈ *T*_*k*_. The GLM for ***α***_*k*_ is given by


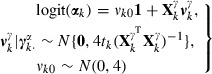
(3)

where logit(***α***_*k*_) is the vector of components logit(*α*_*kt*_) for all *t*. Details on the choice of priors for *ν*_*k*0_ and 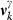 are available in Scheel *et al.* (2011). Specifically, the prior covariance for 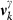 and the variance for *ν*_*k*0_ give unit prior information, as suggested by Ntzoufras *et al.* (2003) and Nott and Leonte (2004) for variable selection in GLMs. The factor 4 is due to our choice of a prior mean of 0 (for a full derivation see Scheel *et al.* (2011)). The prior on ***γ***^*α*^ is exactly the same as the prior on ***γ***^*λ*^, with different hyperparameters 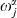.

As stated above, sampling from the posterior distributions can be done separately for the two model parts. For the positive Poisson part, the regression coefficients and the overdispersion variance can conveniently be integrated out from the prior for log (***λ***_*k*_). This avoids varying the dimension of the parameter space (see Appendix A.1). For the positive Poisson intensity, we hence implement a Gibbs sampler. An adaptive Metropolis algorithm (Roberts and Rosenthal, 2006) is implemented for sampling the Poisson rates log (***λ***_*k*_). The algorithms for sampling the variable selection indicator variables ***γ***^*λ*^ and the smoothing parameters ***ω*** follow Smith and Fahrmeir (2007) (using single-site Gibbs sampling for ***γ***^*λ*^); however, the algorithm for ***ω*** is modified to an adaptive Metropolis algorithm. For the Bernoulli component of the model, we implement a reversible jump sampling scheme (Green, 1995) for sampling the regression coefficients 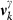 and the variable selection indicator variables 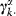 jointly. The algorithm for the smoothing parameters ***ω***^*α*^ is the same as for ***ω***. A graph representation of the complete model and relevant full conditional distributions, as well as other details, are given in Scheel *et al.* (2011).

## 4. Results

To investigate the predictive abilities of our model and to study the fit to the data, we divide the data into a training set, which is used for posterior analysis, and a test set, which is reserved for evaluating predictions. The test set consists of one of the 10 years of data (the year 2001), and the training set contains the other 365×9 days. Posterior analysis is performed via Markov chain Monte Carlo sampling with 10000 iterations after convergence. Trace plots were inspected for signs of lack of convergence. Simulations are set up with two chains and the generalized Brooks–Gelman–Rubin convergence diagnostics (Brooks and Gelman, 1998) are checked for convergence, which appears to be reached. The 10000 iterations took 8 h of running time on a 3-GHz Intel Core 2 processor.

**Fig. 2:**
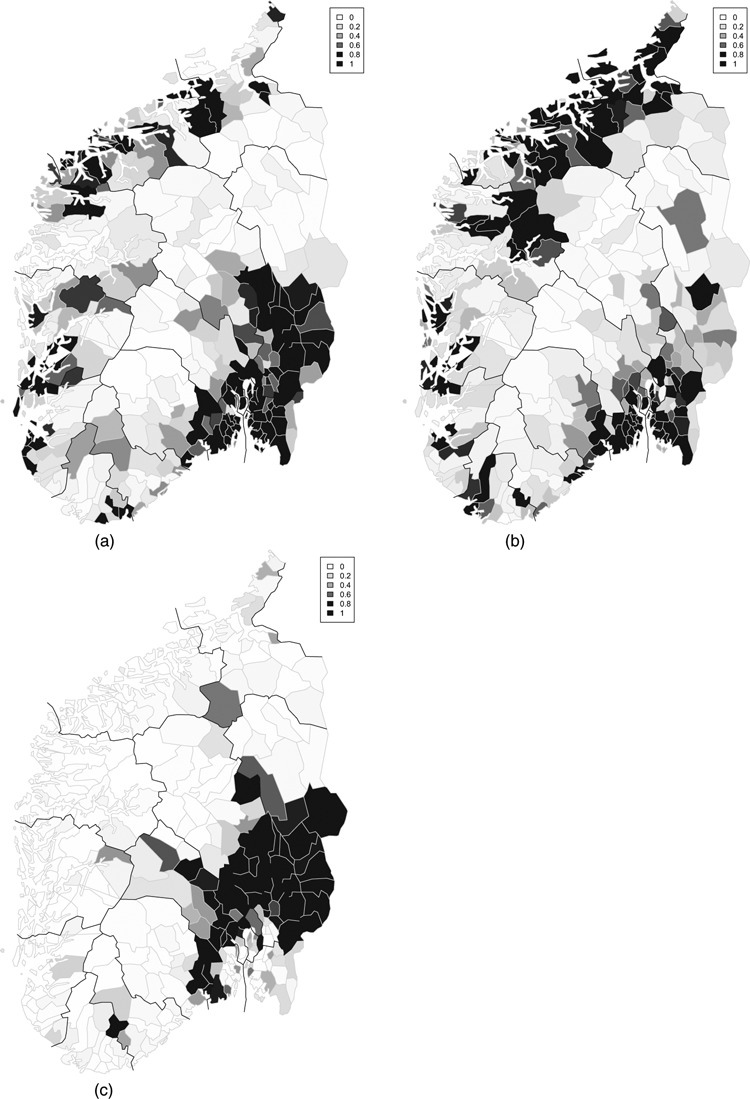
Maps of south and central Norway, divided into municipalities, showing the Monte Carlo estimate of the posterior probability of the binary inclusion variable 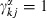 for each municipality *k* for covariate *j*, representing (a) precipitation on the current day *R*_*t*+1_, (b) precipitation on the preceding day and early morning *R*_*t*_ and (c) drainage run-off *D*_*t*_

What are the covariates that appear *a posteriori* significant in the first component of the hurdle model, concentrating on presence or absence of claims? Of the seven weather covariates (see Table 1), four appear to have no, or very little, effect: temperature *C*_*t*_, snow–water equivalent *S*_*t*_, snow difference *S*_Δ_ and the mean precipitation during the previous 3 days, *R*_*t*3_ (the results are not shown). The other three covariates (drainage run-off *D*_*t*_, precipitation on the previous day and early morning, *R*_*t*_, and precipitation on the same day, *R*_*t*+1_) have an important role. Fig. 2(a) illustrates the effect of *R*_*t*+1_, showing for each municipality *k* the Monte Carlo estimate of the posterior probability of 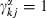. Same day precipitation is important for most of the western coast (though not around the Sognefjord) and in south-east Norway, around the Oslofjord, but less along the south coast, and not at all in the mountainous central areas, where exposure is very low. Further, this effect is present along the southern border to Sweden (south Hedmark). In general, precipitation has less effect in mountainous and remote areas than in urbanized areas, owing to vegetation and soil absorbing water, as opposed to asphalt-covered streets in towns which rely on a properly dimensioned sewage system to collect water run-off. Fig. 2(b) illustrates the effect of precipitation over the preceding day, *R*_*t*_. Comparing these two maps, we see that the effect from the preceding day is stronger and more widespread along the north-west coast, penetrating into the interior of the country, but stops when the altitude begins to increase. The effect of drainage (Fig. 2(c)) is very strong in south-east Norway, just off the coast, and for most municipalities below the mountains. These areas are flatter and water does not escape as easily. Inspecting the posterior probabilities of the models for each municipality shows that the most likely model very often has a probability that is much higher than all other models (an L-shaped density). A map of which model has the highest posterior probability among the 128 possibilities for each municipality can be found in Scheel *et al.* (2011).

**Fig. 3:**
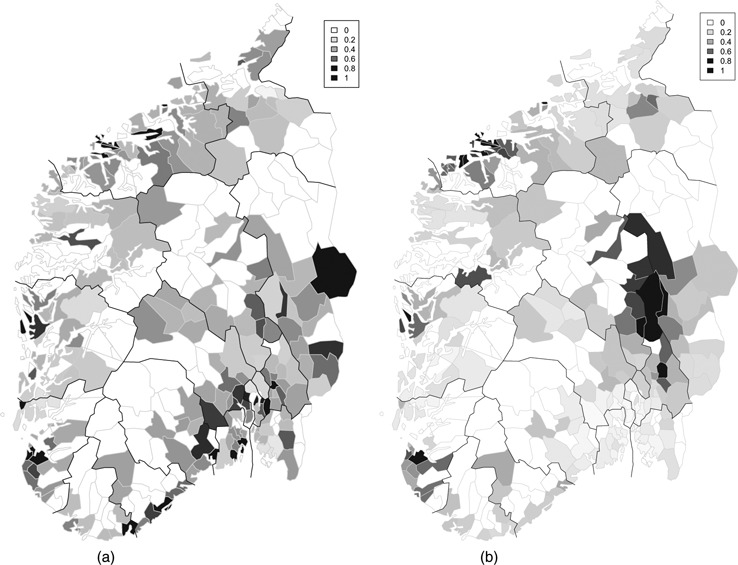
Maps of south and central Norway, divided into municipalities, showing the Monte Carlo estimate of the posterior probability of the binary inclusion variable 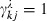 for each municipality *k* for covariate *j*, representing (a) the snow equivalent on the same day *S*_*t*_ and (b) the difference in snow equivalent *S*_Δ_: for 118 of the 319 municipalities, almost all (and in many cases all) positive observed counts equal 1; hence it is not possible to fit a positive Poisson part; the model for these municipalities is collapsed to the binary model of a 0 or 1 count; some municipalities have too few positive counts to fit the full GLM for *λ*_*k*_, and hence only the intercept is included in the *λ*_*k*_-model for these municipalities; for five municipalities, the covariate 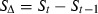 is linearly dependent on the covariate *S*_*t*_ and *S*_Δ_ is therefore not included for these municipalities

Next, we look at the results for the second component of the hurdle model, the positive count, and investigate the posterior probability of 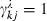. The variables which have large posterior probability of 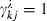 are precipitation on the same day and the preceding day: these maps are very similar to those for the 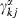
s (the results are not shown). Drainage has no importance for the quantity of claims (the results are not shown), whereas it does have importance for the presence of claims; therefore, it would suggest that it is more associated with isolated weather-related damage. Snow equivalent on the same day, *S*_*t*_ (Fig. 3(a)), and the difference in snow equivalent, *S*_Δ_ (Fig. 3(b)), are important to varying degrees all over the country; *S*_*t*_ shows the highest probability, mostly along the coast, and *S*_Δ_ mostly on the border between the regions Oppland and Hedmark. On the west coast, snow is quite rare. When it snows, the temperatures are mostly around freezing, and the snow is wet and heavy, which can make water collection systems dense. Along the south coast, snow often comes in extremely heavy amounts over short time intervals. A map of which model has the highest posterior probability among the 128 possibilities for each municipality for this positive Poisson count can be found in Scheel *et al.* (2011).

### 4.1. Prediction

We use all the data, except those for 2001, to predict the number of claims for 2001, which is a typical year in our data. The posterior predictive distribution and details on how to sample from it can be found in Appendix A.2. As weather predictions are considered reliable up to 1 week ahead, we study how well we can predict the number of claims in each municipality in each of the 52 weeks of 2001. We use actual observed weather, instead of weather predictions, to compute the posterior predictive distribution for the weekly number of claims for each municipality in 2001.

To evaluate predictive performance, each week of 2001 is classified as one of three types: ‘week type 0’, no claims, ‘week type 1’, 1–3 claims, and ‘week type 2’, four or more claims (nationwide, this is approximately 5% of the weeks). The type of each week for 2001 for each municipality is predicted as the type with the highest posterior predictive probability using the posterior predictive distribution of the number of claims. On average, the percentage of the 52 weeks in 2001 with predicted class equal to observed class for a given municipality is 89%. The ‘success’ percentages for the four largest cities are Oslo and Bergen, 69% of weeks, Trondheim, 71% of weeks, and Stavanger, 67% of weeks. With 46%, Sarpsborg is the only municipality with less than 50% of weeks with a predicted class equal to the observed class.

To investigate how the predictions perform in extreme situations, we consider for each municipality the four weeks among all 52 weeks in 2001 with the highest observed number of claims, i.e. the four weeks with the maximum observed values of 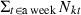. The posterior predictive median with a 95% prediction interval of 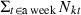, together with the observed number of claims, for those four weeks for Oslo and Bergen are shown in Table 2. There is a tendency to underpredict the number of claims. For comparison, Table 2 also displays the corresponding prediction results for the four weeks with medial observed number of claims: the predictions are excellent, with no sign of systematic bias. A different comparison is described in the bottom half of Table 2. Here, we consider the four weeks among all weeks in 2001 with maximum total precipitation, along with the four weeks with medial total precipitation. Comparing the prediction results for the most rainy weeks with the prediction results for the medial rainy weeks in Bergen and Oslo, we see less evidence of negative bias than for the comparison between the prediction results for the weeks with the maximum and medial observed number of claims. Our model can apparently cope reasonably well with extreme precipitation events but is less able to predict extreme numbers of claims. One possible reason may be that we lack one or more weather covariates that cause extreme numbers of claims.

**Table 2:** Posterior predictive median, 95% prediction interval and actual observation of 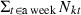 for (a) the four weeks with the maximum observed 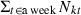, (b) the four weeks with the median observed 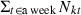, (c) the four weeks with maximum total precipitation and (d) the four weeks with medial total precipitation, for Oslo and Bergen

*Period*	*Results for Oslo*			*Results for Bergen*		
		
	*Median*	*95% prediction interval*	*Observed* Σ*N*_*kt*_	*Median*	*95% prediction interval*	*Observed* Σ*N*_*kt*_
(a)	4	(0, 14)	11	3	(0, 8)	7
	4	(1, 11)	11	3	(0, 7)	7
	3	(0, 8)	8	2	(0, 6)	6
	3	(0, 7)	7	2	(0, 7)	6
(b)	3	(0, 8)	3	2	(0, 7)	2
	3	(0, 7)	3	2	(0, 7)	2
	3	(0, 7)	3	3	(0, 8)	2
	3	(0, 7)	3	2	(0, 6)	2
(c)	5	(1, 13)	5	4	(0, 10)	5
	4	(0, 14)	11	4	(0, 12)	1
	4	(0, 11)	6	3	(0, 9)	3
	4	(1, 11)	11	3	(0, 9)	3
(d)	3	(0, 8)	6	3	(0, 7)	0
	3	(0, 8)	3	3	(0, 6)	2
	3	(0, 8)	1	2	(0, 7)	3
	3	(0, 8)	3	3	(0, 6)	3

**Fig. 4:**
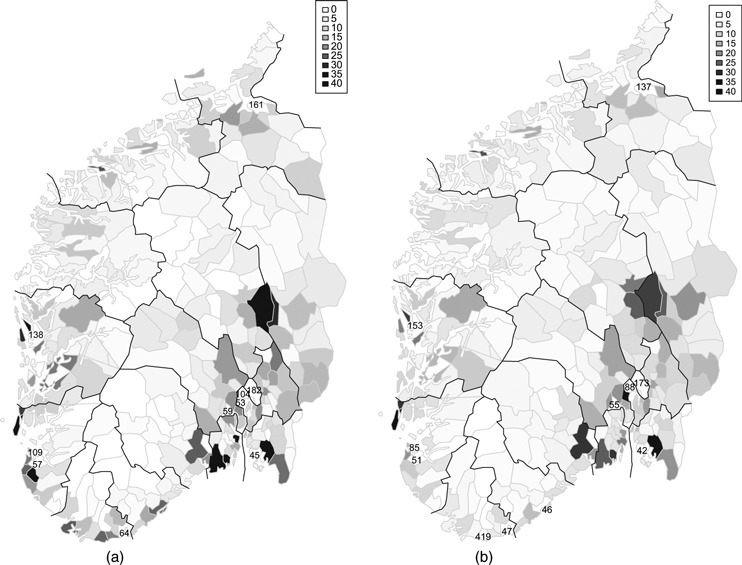
Maps of south and central Norway, divided into municipalities, showing (a) the observed and (b) the posterior predictive median of the yearly number of claims for 2001: for visual reasons in both (a) and (b) counts greater than 40 are marked with the count number; the counts for the large cities are (a) Oslo, 182, Bergen, 138, Trondheim, 161, and Stavanger, 109, and (b) Oslo, 173, Bergen, 153, Trondheim, 137, and Stavanger, 85

Fig. 4 shows maps of the observed and the posterior predictive median of the yearly number of claims for 2001. The observed and predicted yearly counts agree quite well all over the country, with a few exceptions. For the large cities with the most claims, the results are good. The posterior prediction intervals for the yearly number of claims for the four largest cities are Oslo, (142, 207), Bergen, (128, 184), Trondheim, (106, 175), and Stavanger, (66, 108); all include the corresponding observed values.

### 4.2. Sensitivity analysis

The reported results are based on the unit information prior choices for the scale factors of the covariance matrices for 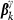 and 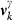, i.e. 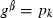 and 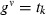. The results may be sensitive to these choices; therefore we also apply the benchmark prior of Fernandez *et al.* (2001), and also multiplying the original choices by 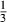 (i.e. *g*^*ν*^≍1000) and 3 (i.e. *g*^*ν*^≍10 000). The results turn out not to be very sensitive to the choice of scale factors. There is a tendency that less covariates are selected for larger values of *g*^*β*^ and *g*^*ν*^. For example, for *R*_*t*+1_, the number of municipalities that have posterior probability of 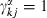 less than 0.5 is 164 for *g*^*ν*^≍1000, 189 for 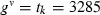 and 200 for *g*^*ν*^ ≍ 10 000. This tendency is in agreement with established theory; small scale factors tend to favour saturated models and large scale factors tend to favour sparse models; see for example George and Foster (2000). Very large values may even result in Bartlett's paradox (Bartlett, 1957), which means that the empty model is favoured. However, looking to predictions, the results are remarkably stable for varying values of *g*^*β*^ and *g*^*ν*^. The results in Table 2 and Fig. 4 are almost identical for all values of *g*^*β*^ and *g*^*ν*^.

## 5. Discussion

This paper develops a new statistical approach to explain and predict insurance losses based on weather events on a local scale. We consider the number of claims; however, similar models can be derived for the type of damage and its economic value. In this case, mixed gamma models are used (Yip and Yau, 2005), conditioned on damage happening (*N*_*kt*_>0). In our model, we separate the occurrence of claims, by municipality and day, from the actual number of claims therein. Results indicate that there are differences in which weather covariates explain and best predict these dynamics; however, these differences may be partly because less data are available to fit the model for the positive counts. We suggest a Bayesian spatially smoothed variable selection approach, assuming local spatial homogeneity in the underlying meteorological causes. As is usual in spatial Bayesian inference, this strengthens inference and prediction, as areas with less data can borrow strength from neighbouring areas with more data. Incorporating geographic gradients to improve neighbourhood structure is possible. We smooth the latent variable selection variables ***γ***^*α*^ and ***γ***^*λ*^, not the regression coefficients. We do not expect the regression coefficients to be smooth in space, as the strength of the effect of the covariates depends more on local factors.

Our study shows interesting regional patterns in vulnerability of buildings to weather covariates. This information may be of interest in the establishment of appropriate building guidelines, as mitigation and prevention are important for the insurance industry. The model can also be used for improving the pricing of policies. Our short-term prediction results are promising; on average we predict the right quantity of claims per week in a municipality in 89% of weeks, using observed weather. This indicates that short-term weather forecasts can be combined with our model to predict high risk situations for damage to structures in Norway. This allows for preventive measures or more efficient dispatch of insurance inspectors. Weather events, which are defined as complex interactions of the weather covariates, should be further investigated as potential cause of damage.

For comparison, we ran the model without spatial smoothing (maps can be seen in Scheel *et al.* (2011)). As expected, the results that were obtained with the full spatial model are more smooth than when running without the hidden Ising fields. In some places, more covariates are selected in the spatial model, thanks to borrowing strength effects from neighbouring municipalities, although they do not enter the reduced model without smoothing. However, mostly the opposite happens, i.e. more covariates are selected in the reduced model compared with the full spatial model. This is because spatial smoothing favours the posterior probabilities of *γ*=1 being close to 0, if this is what neighbouring municipalities tend to indicate. A close inspection of the maps obtained with the models with no spatial component shows that the selected variables vary too much compared with known precipitation patterns and building traditions, which are more smooth in space. This indicates that the Ising-model-modulated variable selection acts in a useful way.

On a broader perspective, our study can be combined with future climate predictions to estimate possible changes in the frequency and number of claims (Haug *et al.*, 2008, 2011). Downscaled regional climate models provide scenarios of temperature, precipitation and many other weather variables at a fine scale (e.g. 10 km × 10 km) for decades to come, under various hypotheses. We can use these climate predictions as input in our model to predict, for example, the distribution of the yearly number of claims in every municipality in the future. The reliability of regional climate prediction is, however, unclear, especially in countries such as Norway which have important mountain chains (Orskaug *et al.*, 2011). These are poorly represented in the rough global circulation models which deliver the input for downscaling. We are currently working on calibration of downscaling techniques. When reliable downscaled climate predictions at a fine scale are available, we shall use them to provide the insurance industry with predictions of exposure to climate risk. Since the underlying statistical distribution of events will change, this could lead to new adaptation strategies for the insurance industry.

## Appendix A

### A.1. Alternative prior specification for log(***λ***_*k*_)

From model (2), we can integrate out the regression coefficients and the overdispersion variance to obtain the following prior specification for log (***λ***_*k*_):

(4)

where ,  is the number of non-zero regression coefficients and

where  is the average of the elements of ***θ***_*k*_ and

See Scheel *et al.* (2011) for a full derivation of expression (4). This is a more convenient prior specification for log (***λ***_*k*_) than model (2), as it avoids varying the dimension of the parameter space for the positive Poisson model part.

### A.2. Posterior predictive distribution

As described in Section 3, because the zero count part and the positive count part of the model are conditionally independent given the data *N*_*kt*_ we may treat the two model parts separately when performing posterior sampling. However, not conditioning on *N*_*kt*_, the two model parts are not independent, and hence, for predictive sampling of random *N*_*kt*_, the two model parts cannot be treated separately.

Let  be the set of days *t* for which we wish to predict *N*_*kt*_, ,  and  the vectors of *N*_*kt*_, *ζ*_*kt*_ and *α*_*kt*_, . Denote by  the subset of days in  with positive counts in municipality *k*, i.e. , and let  be the number of days in , i.e. . Now,  and  are vectors of log (*λ*_*kt*_) and log (*A*_*kt*_), ,  is the covariate matrix for municipality *k* for  and  is the submatrix of the test data covariate matrix corresponding to the days . Also, denote here by  the submatrix of the training data covariate matrix corresponding to the days with positive count . The columns of  are ‘centred and scaled’ by using the same centre and scaling factors as used to produce the training covariate matrix **X**_*k*_ (which is used for the posterior analysis). Similarly, the columns of  are ‘centred and scaled’ by using the same centre and scaling factors as used to produce . To ease notation, in what follows we drop the municipality index *k* on the covariate matrices.

The posterior predictive distribution of  (for ) for a municipality is given by

where

The posterior predictive distribution is conditional on the observed data **N**_*k*_ for *t* ∈ *T*_*k*_, which do not include the days  for which we want to predict *N*_*kt*_. A derivation of the full conditional  is shown in Scheel *et al.* (2011).
